# Exploring the contagion routes of digital emotion on health topics: A multidimensional configuration analysis pre-post rumor refutation

**DOI:** 10.1371/journal.pone.0352675

**Published:** 2026-08-05

**Authors:** Huawei Zhi, Yan Qiao

**Affiliations:** School of Economics and Management, Tiangong University, Tianjin, China; Department of Technical Education, Training and Skill Development, West Bengal, INDIA

## Abstract

Health topics are consistently prevalent on social media and are highly susceptible to rumor. The effectiveness of rumor refutation in fostering positive shifts in public opinion remains to be empirically validated. On social media platforms, digital emotion contagion significantly influences netizens’ attitudes toward health topics and the reception of information aimed at rumor refutation. The Weibo platform is employed to gather extensive user interaction data, and authoritative rumor refutation information is selected as key nodes. Based on digital emotion contagion theory and tipping point theory, this study examines the multidimensional factors influencing digital emotion contagion in the dissemination of health topics. Sentiment analysis (SA) and social network analysis (SNA) are employed to assign values to variables. This study performs a multidimensional configuration analysis of the digital emotional contagion process pre- and post-refutation. Prior to refutation, topic type triggers positive digital emotions among rational netizens, whereas opinion leaders are more likely to elicit such emotions among conformist netizens. When opinion leaders are present and network density is high, negative digital emotions are more likely to propagate. The effectiveness of official institutions in rumor refutation is determined by various factors, including topic type, the role of opinion leaders, the emotional tone of the content, and network density. These factors collectively help suppress the dissemination of negative digital emotions. Research suggests that official refutations of rumors do not always lead to an improvement in digital emotional states; rather, the more prevalent a health topic becomes, the more pronounced the effect of refutation. When the topic is widely disseminated and the audience’s values are aligned, refutation can positively influence digital emotions. An expandable analytical framework is offered, establishing a new foundation for investigating how to promptly correct rumors and effectively guide digital emotions during the dissemination of health topics.

## Introduction

In a highly interconnected online information environment, public health topics are highly likely to attract widespread attention on social media platforms, as they directly concern the survival and safety of the public [1]. However, high levels of public attention are often accompanied by the fragmented dissemination of information and the proliferation of online rumors In discussions of such crisis topics, netizens show a distinct tendency to favor the spread of highly emotional information [2]. As information technology advances, digital emotion is defined as an emotional state conveyed and engaged with via digital media (e.g., text, emojis, multimedia) [3]. It is worth noting that social media platforms influence the contagion dynamics of digital emotions through multiple mechanisms. Existing research has shown that these mechanisms encompass at least the following three aspects: First, in pursuit of user engagement, social media platforms often manipulate information visibility through algorithms, artificially amplifying the frequency and intensity of users’ exposure to emotional content [4].Next, the design of social features such as likes and comments lowers the threshold for emotional expression and contagion, while aggregation functions consolidate scattered emotional expressions into visible collective emotions [5]. Finally, the real-time interactive features provided by platforms create a sense of co-presence, prompting users to exhibit conformity and synchronization effects in emotional expression [6]. These platform-mediated intervention mechanisms, combined with the inherent immediacy and network structure of social media, not only accelerate the spread of health rumors but also give rise to the phenomenon of digital emotion contagion. Substantial evidence suggests that users’ emotional tendencies influence those of other users, thereby affecting netizens’ attitudes and subsequent behaviors toward health events on social media [7]. Digital emotion contagion on social media differs from face-to-face emotion contagion; it is a form of emotion contagion mediated by social media platform operators [8]. Therefore, investigating the mechanisms of digital emotion contagion in health-related emergencies has become a major practical challenge for current cyberspace governance. When health topics are widely discussed on social media, users are reluctant to invest additional time in verifying the authenticity of posts [9]. The spread of false news not only causes confusion but also provides incorrect advice, thereby misleading people’ s behavior and even endangering lives [10]. Does timely rumor refutation by public health authorities necessarily restore netizens’ digital emotions to a rational state? Existing research on the effectiveness of rumor refutation has primarily proceeded along three lines of inquiry, each of which has its own limitations. First, some scholars argue that official institutions, by providing accurate information, can correct erroneous cognitions to quell public opinion and curb the spread of negative emotions [11,12], even treating rumor refutation as the end point of emotional evolution [13]. However, this perspective overlooks the moderating role of emotional tone of content on user information processing in the social media context. Second, motivated reasoning suggests that people tend to accept information consistent with their own positions. When rumor refutation challenges existing cognition, it triggers defensive processing, leading individuals to reject or counter the corrective information [14]. The release of rumor refutation information may inadvertently reinforce the rumor by restating it, thereby strengthening people’s memory of the rumor and increasing their belief in it [15]. This is known as the backfire effect, which represents an extreme manifestation of motivated reasoning in specific contexts [14]. However, most experiments are conducted in controlled environments, leaving a gap in research on emotion contagion within real-world social networks. Third, research grounded in emotion contagion theory has confirmed that digital emotions spread among users through social networks [4]. However, these studies have not incorporated rumor refutation as a key intervention node into their analytical frameworks. In summary, the existing literature lacks an integrated theoretical explanation and empirical testing regarding the intersecting issue of digital emotion contagion pathways pre- and post-refutation interventions. At the methodological level, existing research on rumor refutation effectiveness has predominantly relied on regression analysis and experimental designs, focusing on identifying the net effect of single variables. Consequently, such approaches struggle to capture the equivalent pathways that may arise from the synergistic effects of multiple factors. Social phenomena are often driven by specific combinations of multiple conditions rather than by the linear superposition of single factors [16]. Recent applications of the fsQCA method in the field of information dissemination have shown that configuration analysis can effectively reveal complex causal mechanisms. However, this method has not yet been systematically applied to the study of digital emotion contagion in the context of rumor refutation. The above research reveals that digital emotion contagion is not the result of independent variables acting in isolation, but rather a complex product of the interplay and synergy among multiple dimensions, including micro-level emotional expression, meso-level network structure, and macro-level group cognition. How to systematically reveal the combinatorial effects of these factors is a pressing research question that needs to be addressed.

In terms of basic theoretical choices, existing research has tended to adopt traditional epidemic models to analyze the process of digital emotion contagion. These studies focus primarily on transmission speed, infection direction, and duration, while overlooking the influence of social structure and group interaction [17,18]. However, as pointed out by Wrobel et al. (2019), although the spread of digital emotions shares characteristics similar to virus transmission, it is not a process of automatic infection but rather a social cognitive process shaped by the joint effects of platform mechanisms, network structure, group identity, and external intervention [19]. This perspective provides a direct theoretical basis for this study to move beyond traditional models. On this basis, tipping point theory is introduced as the core theoretical framework. This theory not only aligns with the epidemic nature of emotion contagion but also more comprehensively integrates the synergistic effects of the communicator, communication content, and communication environment on emotion contagion from three dimensions: the law of the few, the stickiness factor, and the power of context [20]. Consistent with the multi-dimensional theoretical framework, this study methodologically overcomes the limitations of traditional single‑cause analysis and creatively integrates sentiment analysis (SA), social network analysis (SNA), and fuzzy set qualitative comparative analysis (fsQCA) into an organic whole (SA-SNA-fsQCA). This study takes 12 health related events from the Zhiwei Event Insights platform as samples and collects 160,000 comment data entries from Weibo. It accurately measures netizens’ emotions at the micro level of sentiment, analyzes the structure of interaction networks at the meso-level, and ultimately employs macro-level configuration analysis (fsQCA) to explore the compound mechanisms through which different combinations of condition variables influence negative digital emotion contagion pre- and post-refutation.

The purpose of this study is to identify the complex configurations of factors that, in the context of public health topics, lead to the failure of official rumor refutation and the persistent contagion of negative digital emotions. The contributions of this study are mainly twofold. At the theoretical level, this study integrates the tipping point theory with fsQCA, thereby extending digital emotion research from a single linear attribution perspective to a complex configuration perspective. It reveals the differentiated equivalent pathways of digital emotion contagion pre- and post-refutation, thus enriching the theory of emotion contagion in complex information environment; At the practical level, the findings not only precisely identify the high-risk contexts that foster negative emotions post-refutation, but also transcend the conventional cognitive limitation that rumor refutation should rely solely on official information dissemination. Furthermore, they provide a scientifically grounded decision-making basis for multiple stakeholders, including media operators, relevant professional researchers, and public figures, to collaboratively foster a rational communication environment for health issues and to optimize strategies for guiding digital emotions.

## Literature review

### Health topic dissemination

Currently, the research focus on health issues has shifted from political events to public health crises, while related communication research has also transitioned from traditional media to social media platforms [21]. Health topics cover a wide range of areas, including food safety, health and regimen, lifestyles, medical resources, and exhibit diverse characteristics when disseminated on social media [22]. Firstly, netizens demonstrate varying levels of interest in health issues, with the majority of discussions centering on specific concerns, which results in notable imbalances in information dissemination [23]. Secondly, on social media platforms, the dissemination of health topics is characterized by strong interactivity and multidirectional flow. Engagement and dialogue among netizens play a critical role in the effective transmission and reception of information [24]. Finally, when discussing health topics, netizens tend to place trust in and refer to authoritative sources of information, which imbues the dissemination of health topics with an authoritative orientation [25].

The high level of attention and dissemination surrounding health topics has led to a common negative issue, namely the rapid generation and dissemination of rumors [26]. Rumors are unverified statements of information that are widely disseminated [27]. Early studies generally regarded official rumor refutation as an effective means to strengthen emergency response and correct individuals’ cognitive biases toward misinformation [28], as well as to improve public health literacy and information discernment [29]. As research has progressed, some scholars have suggested that in the dissemination of health topics, rumor refutation does not necessarily lead to a reversal of digital emotions [30]. Other scholars argue that people tend to accept information consistent with their own positions, yet when rumor refutation challenges their existing cognitions, its effectiveness is diminished [31]. However, such studies remain confined to single-variable testing, failing to systematically explain why the same rumor refutation produces opposite effects across different communication contexts. They also do not distinguish among diverse health topics, such as food safety, disease, wellness, and personal safety, regarding refutation outcomes, and thus lack an integrative framework to explain the complex causes of refutation failure [32]. Therefore, improving the effectiveness of rumor refutation on health topics is an important issue.

Future efforts need to adopt a configuration perspective and dynamic comparative designs to uncover the deep mechanisms underlying the heterogeneity of refutation effectiveness.

### The tipping point theory

Proposed by Gladwell in 2000, the tipping point theory compares the spread of ideas, products, information, and behaviors to an epidemic, defining the tipping point as the moment that causes a sudden change in trends [33]. Gladwell (2000) identifies the stickiness factor, the power of context, and the law of the few as the three core factors that trigger the tipping point [33]. Tipping point theory has been widely applied in various fields of research, including psychology, anthropology, and sociology [20]. The tipping point theory not only covers the similarities between digital emotion contagion and epidemics, but also comprehensively considers the various factors that influence its spread. Therefore, it is more appropriate to use the tipping point theory for emotion contagion research. In the field of netizen psychology research, the three core elements of the tipping point theory, namely the law of the few, the stickiness factor, and the power of context, are adopted to match the communicator, communication content, and communication environment [20].

The communication content serves as a pivotal factor influencing digital emotions. Primarily, the varying categories of topics propagated across social media platforms exhibit differential impacts on digital emotional responses [34]. In addition, posts with a strong emotional tone of content, especially those from popular users, have a complex effect on users’ digital emotion contagion and may also trigger large-scale emotional resonance, leading to an imbalance in the digital emotional ecosystem [35–37]. The stickiness factor law posits that information, products, or ideas should possess memorable characteristics. The topic category of health rumors directly determines whether the information is likely to be attended to or shared, which constitutes the fundamental source of stickiness. Furthermore, emotional expression enhances the memorability of information, and information that meets the stickiness factor must possess memorable qualities; thus, the two together constitute the stickiness characteristics of the communication content. In summary, both the topic type and emotional tone of contents have a significant impact on netizens’ emotions.

Opinion leaders are defined as individuals who have the ability to influence the views and behaviors of others in their social environment, including neighbors, friends, and people with broad social status [38]. Compared to traditional mass media, opinion leaders play a more prominent role in information communication because they have gained the trust of netizens, and a significant negative correlation exists between trust and negative emotions [39]. In addition, digital emotions are also influenced by the interactions of other netizens’ opinions. The closeness of interpersonal relationships has a significant effect on the contagion of digital emotions; the closer the relationship, the more significant the spread of positive emotions [40]. Social network density refers to the ratio of the actual number of connections between network members to the potential number of connections, which also has an impact on the spread of digital emotions [41]. The power of context holds that human behavior is deeply influenced by the external environment; in other words, the environment acts as a catalyst for behavior. Opinion leaders are key nodes in communication networks and authoritative spokespersons within the environment, capable of guiding the trend of group emotions. Social network density represents the degree of closeness in group interactions; high density networks accelerate emotional closure, forming echo chambers. Together, they characterize the structural features of the communication environment, determine the speed, scope, and degree of polarization of digital emotion contagion, and fully align with the core explanatory power of the power of context. Compared to studies that only focus on static attributes of nodes, it is crucial to select opinion leaders and social network density as measurement indicators in the communication environment.

Group identity is a unique social bond formed among group members based on shared perceptions, attitudes, and interests [42]. This sense of belonging enhances netizens’ emotional expression within the group, making it more likely for them to experience emotions that align with the digital emotions when they are closely connected to the group [43]. Public attention represents the scarce resources that citizens invest in public issue debates, such as time and other resources [44]. Netizens activity significantly influences emotional expression. The higher the activity level, the greater the user’s interest in the topic, further confirming the significant impact of public attention on digital emotions [45]. Therefore, researchers selected group identity and public attention as variables to explore their role in the digital emotion contagion effect. The law of the few posits that communication is driven by key groups, and that their participation motivation, sense of belonging, and attentional investment determine whether emotions can be continuously amplified and become prevalent. A sense of belonging and value consistency are the core driving forces behind group collaborative expression and same direction emotion contagion. Netizen attention also represents group attentional investment and willingness to participate. Together, they characterize the psychological and behavioral features of the communicator group, corresponding to the mechanisms by which the law of the few drives emotional outbreaks. Although these variables have been confirmed to be associated with emotions, existing research mostly focuses on independent main effects, neglecting multidimensional synergistic mechanisms, and there is a clear gap regarding the interactive effects of variables.

### Digital emotion contagion

Digital emotion contagion, as a media-mediated emotion contagion phenomenon, is influenced by digital media platforms, which enhance netizens engagement by amplifying the frequency and intensity of emotional experiences [4]. Positive digital emotion contagion can encourage netizens to adopt healthier lifestyles, whereas negative emotion contagion may lead to heightened fear and anxiety regarding health topics, thereby influencing their health-related behaviors [46]. When exploring how to intervene in and reverse such emotional evolution, the conclusions of existing literature show a clear polarization: On the one hand, refutation as an intervention can change the direction of digital emotion contagion. For example, when official institutions refute rumors by presenting factual truth, people’s negative emotions are alleviated, enabling them to respond to the rumor more positively [47]. On the other hand, when the means of refutation do not meet practical needs, the impact of rumors not only fails to diminish but also deepens and widens opinion divisions and misunderstandings [48]. These contradictions indicate that existing linear models can no longer explain the complex boundary conditions of rumor refutation. In other words, identifying which combinations of information, context, and actors lead to the failure of refutation and the intensification of negative emotions is a core gap that urgently needs to be addressed in the current governance of digital public opinion.

Existing studies have estimated digital emotion contagion through the intensity of emotions expressed in netizens’ comments [49]. Most researchers use binary methods to classify digital emotions and study the interaction and stability between negative and positive emotions [50]. Therefore, this study uses the dichotomous method to assess the intensity of digital emotion contagion.

This study integrates emotion contagion theory, tipping point theory, and health rumor refutation to explain the complex phenomenon of digital emotion evolution under refutation intervention. Specifically, emotion contagion theory reveals the psychological mechanisms of emotion contagion between individuals [51]. However, this theory focuses on the micro-level process of emotion contagion and lacks examination of the macro communication context [52]. Rumor refutation research focuses on the impact of corrective information on false beliefs [12]; however, most studies only consider the effect of the refutation information source, neglecting the emotional chain reactions triggered by interactions among netizens [53,54]. Tipping Point Theory provides an integrative framework that explains the mechanisms of outbreak of social phenomena through three dimensions, the law of the few, the stickiness factor, and the power of context, yet its application in the field of digital emotion contagion remains in an exploratory stage. In the existing literature, although some scholars have attempted to integrate emotion contagion with network structure or explore the psychological mechanisms of rumor refutation [55–57], few studies have simultaneously examined how refutation, as an external intervention, reshapes the path of digital emotion contagion by altering communicator influence, information stickiness, and the network environment. Bail et al. (2018), in their study on political polarization, pointed out that the complexity of emotion contagion requires the integration of multiple theoretical perspectives; however, this call has not yet been answered in the field of health rumor refutation [58]. Therefore, the theoretical integration in this study is not a simple patchwork of concepts, but a necessary theoretical innovation addressing the specific phenomenon of emotional evolution driven by multiple factors under refutation intervention.

In summary, this study aims to break the explanatory limitations of single variable attribution. Based on tipping point theory, it investigates the configurational equifinal effects of topic type, emotional tone of post content, opinion leaders, social network density, group identity, and netizen attention on digital emotion contagion.

### Analytical framework

**Fig 1:**
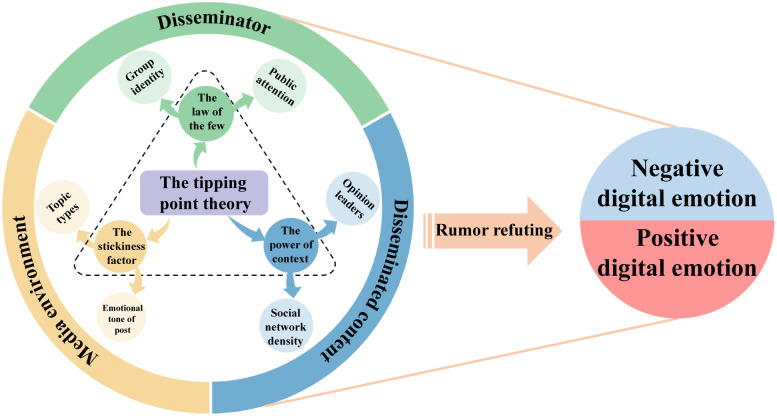
An analytical framework of digital emotion contagion.

Through the integrated application of three research methods, namely sentiment analysis (SA), social network analysis (SNA), and fuzzy set qualitative comparative analysis (fsQCA), this study achieves a systematic analysis of the phenomenon of digital emotion contagion at the micro-emotional, meso-structural, and macro-mechanism levels. Through theoretical innovation and methodological integration, this study not only characterizes the equivalent configurational network of multidimensional variables pre- and post-refutation but also provides an extensible analytical framework. This framework can be applied to a broader range of issues, supporting collaborative refutation, guiding positive emotions, and building a healthy and resilient public opinion ecosystem. The theoretical framework of variables in this study is shown in Fig 1.

## Methods

### Research design

The data collection and analysis process of this study is divided into four main stages. During the case selection and division stage, this study employed the “Zhiwei Event Insight” platform to collect 12 influential case events and categorized them accordingly. The “Zhiwei Event Insight” database is an internet-based platform specializing in aggregating and presenting social hotspot events, developed and operated by Zhiwei Data. To ensure that the cases can fulfill the design objective of pre- and post-refutation comparison in this study, the selected cases meet the following criteria: First, the events must strictly belong to the public health domain, specifically including four subcategories: food safety, disease, health and regimen, and personal safety, to ensure diversity in topic types; second, the events must have a clear two-stage structure, namely a rumor diffusion stage and an official refutation stage by authoritative institutions, with an identifiable temporal boundary between the two stages; finally, it is ensured that each case has sufficient data volume in both the pre-refutation and post-refutation stages to satisfy the measurement of all variables. On this basis, this study establishes the following exclusion criteria: To begin with, cases with a time interval of less than 24 hours between the rumor and refutation are excluded, because such a short time window is insufficient to form an independent emotional evolution stage. Next, cases where the source of refutation information is a non-official agency or non-involved parties are excluded to ensure the consistency of authority of the refutation information. Finally, cases with a total number of comments below 2,000 are excluded to ensure the statistical reliability of social network analysis. Following the above selection criteria, 12 cases were finally identified from the health-related events recorded in the “Zhiwei Event Insight” platform between 2019 and 2024. This study uses the complete statement issued by official agencies or the parties involved in the rumor as the marker of refutation, dividing the case set into two groups: pre-refutation and post-refutation. The time demarcation point follows the following operational rule: It is defined as the first release of a complete statement by an authoritative agency or the parties involved in the rumor, which contains a clear denial of the rumor content and provides factual evidence. All relevant comment data before this time point are classified into the pre-refutation dataset, and comment data after this point are classified into the post-refutation dataset. If multiple refutation statements exist for the same event, the time of the first statement that meets the above criteria is used, because the impact effect of the first authoritative refutation on public opinion is the most significant [59].

During the case data collection stage, this study employed web crawling technology to obtain comment data corresponding to each event pre- and post-refutation on the Weibo platform. This data included user nicknames, published titles, published content, published times, number of comments, as well as first-level and second-level comments under each post. A total of 160,000 data points were collected. All data collected in this study are publicly available and are solely used for academic research. The processes of data collection, storage, cleaning, and analysis fully comply with relevant Chinese laws and regulations on network information, academic ethical norms, and platform access standards. The data cleaning is mainly divided into the following steps: Step 1: Remove invalid data. Delete comments that contain only emojis, only image links, or system-generated reposting phrases that lack substantive textual content. Step 2: Remove duplicate data to ensure the accuracy of emotion measurement results. Step 3: Remove irrelevant data, including advertisements, marketing promotions, and comments that are clearly unrelated to the event topic. Step 4: Remove extreme outliers. Delete long comments exceeding 500 characters per comment, as such texts are mostly reposted news rather than expressions of personal emotion. After the above four-step cleaning process, approximately 160,000 valid comment records were retained.

During the sentiment analysis validation phase, this study comprehensively employed SNA, the BosonNLP sentiment dictionary, Python programming language, and descriptive statistical analysis to identify the key factors influencing digital emotion contagion. Drawing on the tipping point theory, this study developed a digital emotion contagion analysis model and conducted an in-depth examination based on it.

In the empirical analysis and results discussion phase, this study applied fsQCA to jointly evaluate the pre- and post-refutation data, yielding interim conclusions. Based on the above research findings, this paper proposes corresponding strategic recommendations and final conclusions.

**Fig 2:**
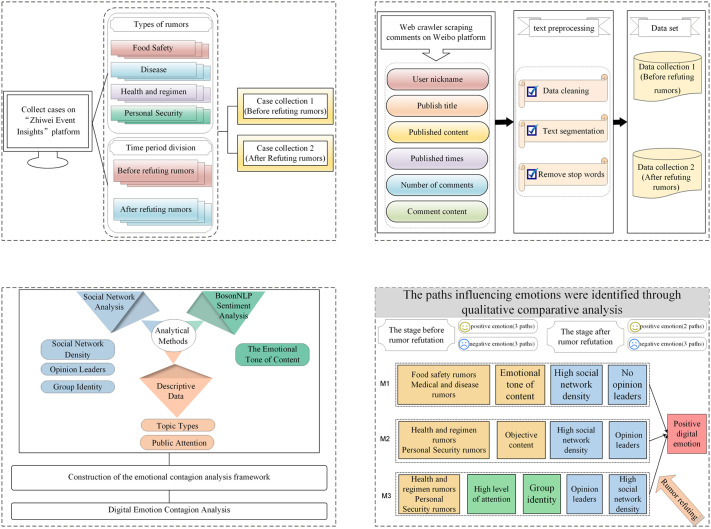
Framework of analytical procedures.

The four research procedures, including case and data collection and collation, variable measurement, and result analysis, are presented in Fig 2.

### SA-SNA-fsQCA

To analyze the contagion routes of digital emotions, this study integrates affective computing sentiment analysis (SA), social network analysis (SNA), and fuzzy-set qualitative comparative analysis (fsQCA) in a systematic and coherent manner. From the micro-level of individual emotions, through the meso-level of network structures, to the macro-level of underlying mechanisms, this study systematically elucidates the mechanisms through which digital emotions propagate and evolve within complex information ecosystems.

Sentiment analysis(SA) is typically defined as a classification task, where each classification type and category is characterized by a sentiment [60]. A systematic literature review study pointed out that sentiment analysis plays an important role in determining the emotional intelligence and emotion contagion of online users [61]. Social network analysis methods are used to explore the interaction patterns of social actors in networks, aiming to analyze relationship content and patterns to understand the connections between actors [62]. The features of social media platforms compared to traditional media include immediacy [63], fragmentation of information [64], and the grid-like structure of the network [65]. In social media, users quickly build a multidimensional network of topics and emotional exchanges through interactive features such as posting and commenting [66]. Digital emotion contagion stems from netizen interactions; thus, social network analysis can reveal interaction patterns and key influencing factors. Sentiment analysis and social network analysis together allow multidimensional examination of factors influencing digital emotion contagion. Additionally, fsQCA is used to explore how multiple routes jointly influence digital emotion contagion. This method not only avoids the limited explanatory power of complex causal relationships between multiple conditions and outcomes but also utilizes the sufficiency and necessity of small-sample analysis conditions as sufficient conditions [67,68].

### Case selection and stage division

In fsQCA, the selection of cases must satisfy the criteria of similarity and diversity, with the aim of achieving a balance between homogeneity and variability. This principle was consistently followed during case selection. Cases were screened from the “Zhiwei Event Insight” database and supplemented with Weibo data to enhance diversity and representativeness. The selected cases were determined according to the following criteria. Typicality: The selected cases represent common health-related rumors, thereby enhancing the applicability of the conclusions. Diversity: This study included rumors related to food health, diseases, health and regimen, and personal security, aiming to provide a comprehensive perspective on the issue. Certainty: Only cases with clear, well-documented, and complete event timelines were considered to ensure the reliability of the conclusions. Comprehensiveness: The cases selected for this study have sufficient data and background information to ensure detailed analysis. This study selected 12 health-related rumors from 2020 to 2024, as shown in Table 1.

**Table 1:** Typical cases of health rumors.

	Name	Time of the incident	Turning point time
**1**	The blogger’s gastroenteritis is suspected to have been caused by an unknown ingredient in sheep soup	14 December 2024	16 December 2024
**2**	The incident that “Shuanghuanglian oral liquid” can inhibit the novel coronavirus	31 January 2020	1 February 2020
**3**	Starchy sausage is made from mashed chicken bones	15 March 2024	16 March 2024
**4**	Soaking in hot springs may lead to HPV infection	3 January 2024	6 January 2024
**5**	An explosion occurred in Songjiang, Shanghai	29 January 2024	30 January 2024
**6**	The food delivery poisoning incident at Xiangtan University	18 April 2024	19 April 2024
**7**	A fascia gun can treat hemorrhoids	22 November 2023	23 November 2023
**8**	In Chengdu, Cordyceps sinensis is suspected of being injected with mercury	18 May 2023	19 May 2023
**9**	Drinking water while eating is not conducive to digestion.	7 July 2023	8 July 2023
**10**	Instant noodles are junk food	6 April 2023	7 April 2023
**11**	Health products can prevent COVID 19	19 March 2022	20 March 2022
**12**	An explosion occurred in the septic tank of Ocean University of China	12 October 2020	13 October 2020

Note: This table collects data from Sina Weibo and covers 12 public health rumor events occurring between 2020 and 2024.

This study explores how the configuration of digital emotion generation routes differs pre- and post-refutation. Therefore, using the official refutation time released by authoritative institutions on Weibo as the node, the emotional trends pre- and post-refutation are distinguished.

### Variable assignment methods

#### Topic type.

Current research findings indicate that in the online contagion of health-related rumors, topics related to food safety constitute 38.91% of the total, disease rumors account for 34.78%, and content focusing on health preservation and healthy lifestyles represents 16.44% [69]. In contrast, rumors related to personal security receive relatively limited scholarly attention. According to the stickiness factor law of tipping point theory, understandable and memorable information has stronger stickiness, and more popular topics have even stronger stickiness. Therefore, this study codes food safety rumors as 1, disease-related rumors as 0.67, health and regimen rumors as 0.33, and personal security rumors as 0.

#### Emotional tone of content.

Emotional intensity enhances the memorability of information and serves as the core source of stickiness. Studies have found that in public emergencies, information content with strong emotion is more likely to become the focus of public attention than neutral content and can be retweeted more quickly and frequently [70]. Therefore, emotional valence and intensity directly determine the direction and speed of digital emotion contagion. This study employs Boson Natural Language Processing (BosonNLP) technology to compute the emotional valence of text. BosonNLP is one of the commonly used sentiment analysis methods. Its distinctive feature is that the BosonNLP sentiment vocabulary database is a general corpus constructed based on multi-category data such as news, microblogs, and comments, covering emerging online terminology, spelling variations, and more. Since this study collects data from Weibo, using the BosonNLP dictionary and Python programming language for sentiment analysis can improve the accuracy of sentiment measurement. The research process begins with text preprocessing, followed by sentence-level sentiment analysis conducted using the BosonNLP sentiment dictionary and the Python programming language. Finally, the average sentiment score is calculated, and the results are normalized using the absolute value method. Finally, during the data input stage prior to configural analysis, secondary encoding is conducted by integrating both the absolute value of the sentiment score and the semantic emotional orientation, thereby enabling a synchronized representation of digital emotion intensity and emotional direction.

#### Opinion leaders.

Under the power of context, opinion leaders serve as authoritative voices to guide the evolution of collective emotions. Thus, the higher nodal influence opinion leaders possess in networks, the more prominent their impact on emotion contagion. The method for identifying opinion leaders is based on the random walk algorithm, with the most prominent example being the PageRank algorithm developed by Brin and Page. PageRank algorithm not only takes into account the number of external connections when calculating the influence of network nodes, but also incorporates the influence of the connected nodes into the computation [71]. Previous studies have compared the effectiveness of various algorithms in identifying opinion leaders, with results indicating that the PageRank algorithm demonstrates superior performance compared to centrality-based algorithms [72]. PageRank uses an iterative method to solve the problem. When encountering a self-loop or node trap, the netizens will choose to re-select a random web page link with a certain probability α. The formula is as follows [73,74]:


$$\begin{array}{c} PR\left(p_{i}\right)=\alpha +(\text{1}-\alpha )\sum_{P_{j}\in I\left(p_{i}\right)}\;\frac{PR\left(p_{j}\right)}{O\left(p_{j}\right)} \end{array}$$ (1)

Among these, $PR\left(p_{i}\right)$ is the PageRank value of node $p_{i}$ obtained through iterative calculations, $I\left(p_{i}\right)$ is the set of in-degree values for node $p_{i}$, $O\left(p_{j}\right)$ is the total number of out-degree values for node $p_{i}$, and α is the random jump probability, typically set to 0.15. $\sum_{P_{j}\in I\left(p_{i}\right)}\;$ covers all inbound nodes $p_{j}$ pointing to $p_{i}$. $PR\left(p_{j}\right)$ is the PageRank value of node $p_{j}$, while $\frac{PR\left(p_{j}\right)}{O\left(p_{j}\right)}$ is the weight allocated from $p_{j}$ to $p_{i}$.

#### Social network density.

The accelerated emotional closure and echo chamber formation in high density networks embody the power of context. Existing studies explore the impact of interpersonal intimacy on emotion contagion, revealing that closer interpersonal bonds facilitate more prominent positive emotion contagion [40,75]. In dense social networks, individuals engage in more frequent interactions with other network members, leading to a more refined understanding of both themselves and others [75]. This paper establishes a directed network, represented by G=(V,E), where V is the node and E is the set of edge relationships. The formula for measuring social network density is:


$$\begin{array}{c} D=\frac{e}{N(N-\text{1})} \end{array}$$ (2)

D represents network density, e denotes the number of relationships among all nodes, and N represents the total number of nodes in the network. In general, density values range between 0 and 1, with values closer to 1 indicating closer relationships and more active dissemination among members. Values closer to 0 indicate more distant relationships and less frequent dissemination among members.

#### Group identity.

The law of the few posits that communication is driven by critical groups, whose participation motivation and sense of belonging determine whether emotions can be continuously amplified and become prevalent. When individuals gain a sense of belonging within a group, interactions strengthen emotional bonds among members and thereby elevate overall group emotions [76]. Accordingly, higher levels of group identity facilitate more pronounced digital emotion contagion. In social networks, diverse types of subgroups not only shape the interpersonal relationships among members but also exert a significant impact on the overall functionality and operational efficiency of the network. In studies assessing group identity, scholars commonly employ questionnaire surveys and apply a 5-point Likert scale to quantify the collected data. For example, studies may include statements such as, “When the majority of group members hold pandemic-related attitudes that align with my own, I experience a strong sense of belonging.” and “I believe that widespread expression of negative emotions toward the pandemic by users helps protect the rights of vulnerable groups during pandemic-related hot topics” [43]. Once netizens develop a sense of belonging to a group, they form cohesive subgroups with consistent attitudes, perceptions, verbal behaviors and emotional tendencies. The cohesive subgroup coefficient serves as a core indicator for identifying communities with group identity. Chin et al. have empirically verified that this coefficient can act as an effective metric to measure group identity [77]. This is because cohesive subgroups represent the objective manifestation of group identity, and stable reciprocal networks correspond to high levels of belongingness and loyalty among members. Accordingly, it is reasonable to adopt the cohesive subgroup coefficient to measure the intensity of group identity. The formula for calculating cohesive subgroups is as follows:


$$\begin{array}{c} \frac{\text{1}}{n}\sum_{i=\text{1}}^{n}\;\frac{\left|\left\{e_{jk}:\nu_{i},\nu_{k}\in N_{i},e_{jk}\in E\right\}\right|}{k_{i}\left(k_{i}-\text{1}\right)} \end{array}$$ (3)

In this formula, n represents the number of nodes, $e_{jk}$ represents the connection between two nodes $\nu_{i}$ and $\nu_{k}$, $N_{i}$represents the domain nodes of node $\nu_{i}$, and $k_{i}$ represents the number of domain nodes of node $\nu_{i}$.

In this study, Gephi is used to measure group identity with the cohesive subgroup coefficient, identify authoritative opinion leaders based on PageRank scores, and quantify social network density via the network density coefficient.

#### Public attention.

According to the law of the few, collective attention promotes sustained emotional amplification. Emotions spread via active user debates over relevant cases [45]. High user activity indicates great public attention to corresponding topics. At the individual citizen level, if a person is willing to invest valuable time and effort in searching information about public issues online, researchers can reasonably infer that they are interested in and highly concerned about that public issue [78]. This determines whether emotional diffusion reaches the tipping point. Accordingly, this study adopts topic discussion volume to measure attention levels. This study employs the number of discussions under each event topic on the Weibo platform as an indicator of public attention.

#### Digital emotion.

Digital emotion plays a significant role in assessing the emotional intelligence of online users as well as the intensity of digital emotion contagion [61]. This study employs BosonNLP technology to compute the sentiment value of textual content, thereby capturing the emotional tone of content. The research process begins with the preprocessing of post text. Subsequently, the BosonNLP sentiment dictionary, in conjunction with the Python programming language, is employed to conduct sentiment analysis on the text. Next, the average of all scores is computed, and the results are processed using the absolute value method. Finally, during the data input stage prior to the configural calculation, secondary encoding is conducted by integrating both the absolute value of the score and the semantic emotional direction, thereby enabling a simultaneous description of digital emotion intensity and emotional orientation.

### Variable calibration

A core procedure of fsQCA is data calibration, which converts raw data into set concepts and transforms conventional variables into fuzzy variables ranging from 0 to 1 [79]. Referring to previous studies [80], this study adopts the direct calibration method for variable calibration. The 95th, 50th, and 5th percentiles of sample data are defined as the threshold values of full affiliated point, intersection point, and completely unaffiliated point, respectively. This method effectively reduces subjective bias and improves the objectivity and accuracy of calibration results. The calibration anchors of each variable and the calibrated data are shown in S1 and S2 Tables.

## Findings

### Analysis of necessary conditions

Following the calibration procedure, this study conducted a necessity analysis of the conditional variables to determine whether each antecedent variable constitutes a necessary condition for the outcome [81]. Necessity tests were performed on data from both the pre-refutation and post-refutation phases, with the consistency threshold for the tests set at 0.9. Tables 2 and 3 report the necessity tests pre- and post-refutation, respectively. The consistency coefficients for all variables fall below the threshold of 0.9, indicating that none of the individual variables in the research model constitutes a necessary condition for influencing the intensity of digital emotion contagion. The research results align with the theoretical framework of this study, suggesting that the factors affecting digital emotion contagion intensity are systematic and multifaceted.

**Table 2:** Analysis of necessary conditions (pre-refutation stage).

Antecedent condition	Outcome variable positive digital emotion		Outcome variable negative digital emotion	
	Consistency	Coverage	Consistency	Coverage
**Topic Type**	0.72	0.718	0.461	0.463
**~Topic Type**	0.461	0.460	0.719	0.721
**Emotional tone of the context**	0.511	0.535	0.616	0.648
**~ Emotional tone of the context**	0.664	0.712	0.558	0.534
**Opinion Leader**	0.471	0.508	0.669	0.726
**~Opinion Leader**	0.746	0.736	0.547	0.510
**Network Density**	0.448	0.509	0.647	0.738
**~Network Density**	0.769	0.684	0.569	0.509
**Attention of Netizens**	0.555	0.643	0.459	0.535
**~Attention of Netizens**	0.599	0.524	0.694	0.611
**Group Identity**	0.651	0.719	0.446	0.496
**~Group Identity**	0.544	0.494	0.747	0.683

Note: This table presents the results of necessity analysis for variables in the pre-refutation phase.

**Table 3:** Analysis of necessary conditions (post-refutation stage).

Antecedent condition	Outcome variable positive digital emotion		Outcome variable negative digital emotion	
	Consistency	Coverage	Consistency	Coverage
**Topic type**	0.717	0.656	0.454	0.493
**~Topic type**	0.445	0.407	0.683	0.741
**Emotional tone of the context**	0.629	0.589	0.585	0.650
**~ Emotional tone of the context**	0.625	0.559	0.630	0.669
**Opinion leader**	0.470	0.474	0.652	0.782
**~Opinion leader**	0.784	0.655	0.562	0.557
**Network density**	0.424	0.445	0.642	0.801
**~Network density**	0.811	0.656	0.555	0.534
**Attention of netizens**	0.578	0.657	0.530	0.716
**~Attention of netizens**	0.750	0.573	0.746	0.677
**Group identity**	0.617	0.636	0.499	0.610
**~Group identity**	0.622	0.511	0.702	0.685

Note: This table presents the results of necessity analysis for variables in the post-refutation phase.

### Analysis of sufficient conditions

The purpose of sufficient configurations analysis is to assess the explanatory adequacy of different configurations in accounting for the outcome. This study used R software to calculate truth values and refine the truth table, with the raw consistency threshold set at 0.8 to identify configurations associated with the outcome. Due to the small sample size, the frequency threshold was set to 1 [82]. Based on previous studies, this study set the minimum proportion of inconsistency to 0.7 to avoid contradictory configurations [83].

The fsQCA method yields three types of solutions: complex solutions, simplified solutions, and intermediate solutions. Complex solutions cover all possible combinations of conditions, while simplified solutions and intermediate solutions simplify the interpretation [84]. Among the solutions, intermediate solutions and simplified solutions distinguish between core conditions (existing in simplified solutions) and peripheral conditions (appearing only in intermediate solutions) [79]. The analysis is primarily based on intermediate solutions, supplemented by simplified solutions, to identify core and peripheral conditions. Conditions that exist in both intermediate and simplified solutions are considered core conditions. Conditions that appear only in intermediate solutions are considered peripheral conditions.

Analyzing pre- and post-refutation conditions of digital emotion contagion, this study identified four types of configuration sets. These encompass the positive and negative digital emotion configurations in pre- and post-refutation stage. The overall consistency scores of the four sets of configurations reached 0.930, 0.919, 0.808, and 0.889, respectively. The reliability of the conditional combinations as sufficient conditions for the results is relatively high. The overall coverage metrics for the four sets of configurations were 0.588, 0.635, 0.361, and 0.754, respectively. Considering that the public health topics selected in this study span a wide range of industries and that the sample involves network users with highly diverse individual characteristics, the above coverage metrics prove that the explanatory power of the calculation results meets the research expectations.

#### Configurations for achieving positive digital emotions: Pre-refutation.

Pre-refutation, three configurations of positive digital emotions were identified among netizens concerning health topics associated with public health events. The presence of these configurations suggests that even when unfounded or factually inaccurate rumors emerge during the dissemination of public health information, individuals may still maintain a positive and optimistic attitude toward the information released online. Table 4 shows the consistency and coverage levels of the computational results. Pre-refutation, the configurations that evoke positive digital emotions exhibit strong individual consistency (1.000, 0.897, 0.969), confirming their robustness.

**Table 4:** Configurations for achieving positive digital emotion: Pre-refutation.

Conditions	Configurations (positive digital emotion: Pre-refutation		
	BP1	BP2	BP3
**Topic type (T)**	●^a^	●	⊗^b^
**Emotional tone of the context (C)**	•^c^	•	⊗
**Opinion leader (L)**	⊗	⊗	●
**Network density (D)**	⊗	⊗	●
**Attention of netizens (A)**	●	^d^	
**Group identity (I)**		●	●
**Raw coverage**	0.258	0.360	0.240
**Unique coverage**	0.042	0.116	0.185
**Consistency**	1.000	0.897	0.969
**Overall solution consistency**	0.930		
**Overall solution coverage**	0.588		

^a^Core conditions exist.

^b^Core conditions are missing.

^c^Edge conditions exist.

^d^Blank: The conditions have no effect on the result.

In configuration BP1, topic type and public attention are core presence conditions, opinion leadership and social network density are core absence conditions, and emotional tone of content is a peripheral presence condition. The variable configuration suggests that when health topics primarily involve food safety and medical and disease rumors, and when post content or comments exhibit distinct digital emotion signals, netizens tend to rapidly attract public attention and elicit strong positive emotions.

The variable structures of BP2 and BP1 are similar, with group identity replacing public attention as the core presence condition. Differences in individual characteristics of netizens serve as the primary basis for distinguishing between BP2 and BP1. This difference indicates that, when faced with similar health topic content and online channel contexts, capturing netizens’ attention and evoking their resonance have equivalent digital emotion impact effects.

In the BP3 configuration, opinion leadership, social network density, and group identity are core presence conditions, while topic type and emotional tone of content are core absence conditions. This configuration does not include peripheral conditions. The computational results indicate that if the topic content primarily concerns health and regimen and personal safety, and the posts are expressed in a calm and objective manner, even if they fail to attract significant attention from netizens initially, positive digital emotion will rapidly form among netizens with similar values as opinion leaders become involved and netizens engage in close interaction.

#### Configurations for achieving the negative digital emotion: Pre-refutation.

Three configurations were identified that trigger negative digital emotions among netizens pre-refutation. Table 5 shows the high individual consistency scores (1.000, 0.936, 0.878) of the computational results, demonstrating that the condition combinations within these configurations exhibit strong reliability in terms of sufficiency.

**Table 5:** Configurations for achieving negative digital emotion: Pre-refutation.

Conditions	Configurations (negative digital emotion: Pre-refutation)		
	BN1	BN2	BN3
**Topic type (T)**	⊗^a^	⊗	●^b^
**Emotional tone of the context (C)**	^c^	⊗	•^d^
**Opinion leader (L)**		●	●
**Network density (D)**		●	●
**Attention of netizens (A)**	⊗		⊗
**Group identity (I)**	⊗	⊗	•
**Raw coverage**	0.430	0.334	0.239
**Unique coverage**	0.178	0.124	0.080
**Consistency**	1.000	0.936	0.878
**Overall solution consistency**	0.919		
**Overall solution coverage**	0.635		

^a^Core conditions are missing.

^b^Core conditions exist.

^c^Blank: Conditions have no effect on the result.

^d^Edge conditions exist.

The configuration BN1 is composed of three core absence conditions: topic type, public attention, and group identity. This configuration does not include core presence conditions or peripheral conditions. Its structure highlights the advantages of fsQCA in data processing, specifically its ability to provide an error-avoidance type of variable association. Based on the variable assignment principles, it can be observed that if health topics primarily involve health and regimen and personal security, negative emotions may still arise among individual netizens without the need for emotion contagion or accumulation among them.

The BN2 configuration includes two core presence conditions: opinion leaders and social network density, as well as three core absence conditions: topic type, emotional tone of the content, and group identity. Configuration BN2 indicates that when topics related to health and regimen and personal security are presented in a rational and calm manner, if communication context features prominent authoritative opinions and netizens are willing to engage in mutual dissemination, netizens will overlook differences in values, interests, and emotions between one another, gradually developing negative digital emotion spontaneously.

The configuration of BN3 involves a variety of conditional variables, among which topic type, opinion leaders, and social network density are core presence conditions, public attention is a core absence condition, and the emotional tone of the content and group identity are peripheral presence conditions. The computational results indicate that when health topics involve food safety and medical and disease rumors, the deeper involvement of opinion leaders and the more frequent the communication among netizens, the more likely it is to generate negative digital emotions. If a post has a more pronounced emotional tone of content or is provocative, and users participating in the comments have stronger trust or more consistent cognition, this further reinforces the generation of negative digital emotions. Additionally, in such cases, the spread of negative digital emotions is often faster, without a noticeable accumulation process.

#### Configurations for achieving the positive digital emotion: Post-refutation.

Following the release of refutation content by authoritative institutions regarding health-related topics, two equivalent configurations were identified that facilitate digital emotion contagion among netizens, as presented in Table 6. These configurations demonstrate moderate individual consistency scores (0.804, 0.876).

**Table 6:** Configurations for achieving positive digital emotion: Post-refutation.

Conditions	Configurations (positive digital emotion: Post-refutation)	
	AP1	AP2
**Topic type (T)**	●^a^	●
**Emotional tone of the context (C)**	●	⊗^b^
**Opinion leader (L)**	⊗	●
**Network density (D)**	^c^	●
**Attention of netizens (A)**	•	●
**Group identity (I)**	•^d^	•
**Raw coverage**	0.269	0.189
**Unique coverage**	0.172	0.092
**Consistency**	0.804	0.876
**Overall solution consistency**	0.808	
**Overall solution coverage**	0.361	

^a^Core conditions exist.

^b^Core conditions are missing.

^c^Blank: Conditions have no effect on the result.

^d^Edge conditions exist.

The AP1 configuration shows that the core presence conditions for eliciting positive digital emotions in information recipients include topic type and emotional tone of content, while the core absence condition is opinion leaders. Additionally, public attention and group identity function as peripheral presence conditions. Computational results show that for food safety or medical and disease rumors, recipients are more likely to accept official explanations and view health incident handling positively, especially when communicators express strong digital emotions and no opinion leaders comment on official refutations. If the topic receives high public attention and netizens participating in the discussion share similar values, they are more likely to accept the official institution’s handling of public health events.

In the AP2 configuration, topic type, opinion leaders, social network density, and public attention are core presence conditions, while emotional tone of the content is a core absence condition, and group identity is a peripheral presence condition. This combination of conditions indicates that for topics related to food safety and medical and disease rumors, after official institutions refute rumors, as long as netizens participating in topic discussions can interact using rational language, positive emotions are likely to communicate among netizens. If the user group characteristics of those following the topic are relatively concentrated, the efficiency of positive emotion transmission will be higher.

#### Configurations for achieving the negative digital emotion: Post-refutation.

Following rumor refutation, three configurations were identified that may render the refutation ineffective. In other words, negative digital emotion contagion can still occur among netizens. As presented in Table 7, the individual consistency scores of these three configurations are 0.856, 0.880, and 0.950, respectively, demonstrating that the computational results are highly reliable.

**Table 7:** Configurations for achieving negative digital emotion: Post-refutation.

Conditions	Configurations (negative digital emotion: Post-refutation)		
	AN1	AN2	AN3
**Topic type (T)**	^a^		⊗^b^
**Emotional tone of the context (C)**	⊗		•^c^
**Opinion leader (L)**		●^d^	●
**Network density (D)**		●	●
**Attention of netizens (A)**	●	⊗	⊗
**Group identity (I)**	⊗	●	
**Raw coverage**	0.447	0.323	0.356
**Unique coverage**	0.245	0.106	0.154
**Consistency**	0.856	0.880	0.950
**Overall solution consistency**	0.889		
**Overall solution coverage**	0.754		

^a^Blank: Conditions have no effect on the result.

^b^Core conditions are missing.

^c^Edge conditions exist.

^d^Core conditions exist.

The number of conditional variables included in configuration AN1 is relatively small. Public attention is the core presence condition, while emotional tone of the content and group identity are the core absence conditions. This combination suggests that for high-attention health topics, significant variability and calmness among commenting netizens reduce refutation effectiveness, fostering negative digital emotions.

The AN2 configuration includes three core presence conditions: opinion leaders, social network density, and group identity, while public attention serves as the core absence condition. The results indicate that when opinion leaders have significant influence in online social contexts and users with similar values interact frequently, netizens often develop negative digital emotions toward health topics with which they are less familiar. In such cases, rumor refutation does not achieve the desired effect.

In configuration AN3, the three conditional variables—opinion leaders, social network density, and public attention—function in a manner similar to that in configuration AN2. The difference lies in the fact that configuration AN3 emphasizes the influence of topic content on digital emotion following rumor refutation. Topic type functions as a core absence condition, while the emotional tone of content acts as a peripheral presence condition influencing digital emotion. Configuration AN3 indicates that for topics related to health and regimen and personal security, slightly emotional expressions are more likely to lead people to doubt the authenticity or effectiveness of official refutation.

#### Key conditions pre- and post-refutation: Dual roles and dynamic evolution.

Based on the three laws of the tipping point theory, the key conditions affecting digital emotion contagion on health topics play opposite roles pre- and post-refutation, exhibiting causal asymmetry. This phenomenon suggests that official rumor refutation is not merely a simple correction of information, but rather a process that transforms netizens’ truth-seeking state into a game of trust and positions. This shift aligns with the core argument of motivated reasoning, which emphasizes the preservation of pre-existing attitudes, and provides theoretical insights into the backfire effect triggered by rumor refutation. The results further reveal that multiple key conditions exert dual roles pre- and post-refutation, which reflects the dynamic evolution of tipping-point mechanisms across intervention stages.

**Fig 3:**
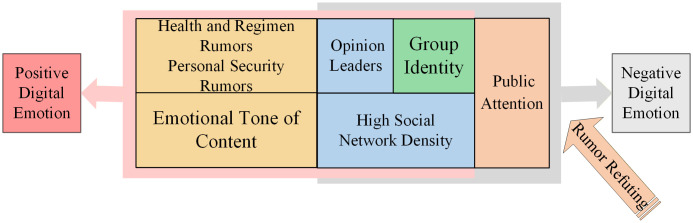
Causal pathways with dual roles pre- and post-refutation.

**Fig 4:**
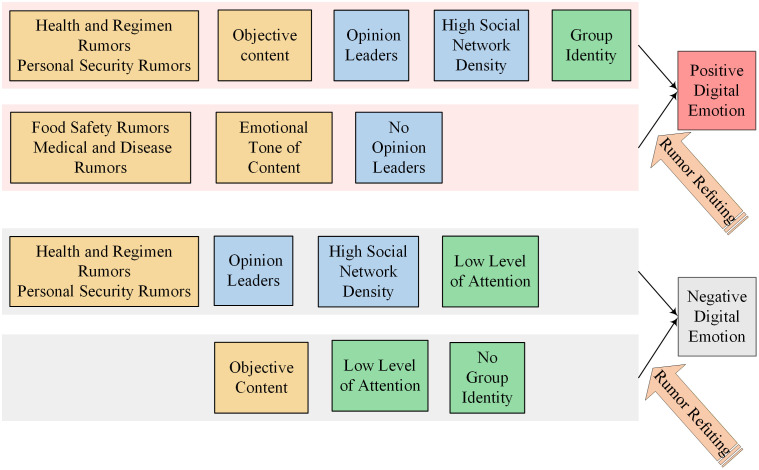
Distinct causal pathways pre- and post-refutation.

The first factor with dual roles refers to opinion leaders in the law of power of context. Prior to rumor refutation, opinion leaders primarily serve a cognitive inspiration function, which alleviates the cognitive load of netizens. Meanwhile, they leverage their online influence to promote consistent emotion contagion; when they deliver positive signals, they tend to catalyze the formation and contagion of positive emotions. However, following official rumor refutation, the role of opinion leaders changes fundamentally. Challenged by such refutation, they may adopt a confrontational stance to safeguard their influence and prior claims, regarding official refutation as evidence of low institutional credibility. Under such circumstances, their influence evokes negative emotions among followers and acts as a catalyst for intensified conflicts (e.g., configurations AN2 and AN3). Social network density complying with the law of the power of the context represents the second factor with dual roles. Prior to rumor refutation, high social network density facilitates resonance among netizens, laying a structural foundation for the diffusion of positive emotions. However, following rumor refutation, this structural effect reverses and accelerates group polarization. It facilitates the emergence of the echo chamber effect and group polarization, accelerating the contagion of negative emotions. This verifies the constraining effect of communication environments on emotion contagion direction. The third critical condition is topic type, which embodies law of the stickiness factors. Configuration results further reveal that the role reversal does not occur indiscriminately, but is moderated by topic type and corresponding information stickiness. Rumors concerning food safety and disease involve broad public interests, enabling easier collective cognition on common issues following rumor refutation. Under such cognition, authoritative refutation information is regarded as a basis for public welfare, which suppresses negative emotional polarization and lays a cognitive foundation for the emergence of positive emotions (e.g., configurations AP1 and AP2). In contrast, for topics with narrow audiences and strong ties to individual identity, such as regimens and personal security perceptions, official rumor refutation tends to be interpreted as a negation of the lifestyles of particular groups. When these specific groups experience perceived threats, group defense mechanisms are activated, intensifying opinion leaders’ confrontational mobilization and opinion polarization, and thereby triggering the post-refutation backfire effect as well as the sustained diffusion of negative emotions (e.g., configurations AN2 and AN3). Fig 3 illustrates the functional pathways of the three dual-role variables pre- and post-refutation within the tipping point theoretical framework. Among all causal pathways, besides those with overlapping antecedent conditions, distinct pathways with entirely different conditions yield identical emotional outcomes pre- and post-refutation, as shown in Fig 4. From the perspective of multi-dimensional synergy, prior to rumor refutation, objective rumor content reduces users’ vigilance. The authority of opinion leaders enhances information credibility, while high density of social networks and group identity further strengthen user trust, ultimately evoking positive emotion. After refutation, this path has become completely invalid, replaced by an entirely new path. After a niche rumor is refuted, users’ trust in authoritative and objective information diminishes, and they become increasingly influenced by emotional content. Even in the absence of opinion leader guidance, positive digital emotions toward the rumor still emerge. At this point, the stickiness factor of emotional tone of content becomes an independent core driving force that sustains emotion contagion. With respect to negative emotions, prior to rumor refutation, under opinion leaders and high density of networks, the negative emotions generated by netizens consist of fear, panic, and anger regarding the threat to their health. Opinion leaders exploit people’s concerns over their immediate interests, transforming them into a sense of crisis; these emotions exhibit strong contagion and action orientation. This also indicates that a negative shift in content influence induces corresponding negative transitions in users’ digital emotions. Post-refutation, the core variables underlying negative emotions shift entirely. Without the involvement of opinion leaders, objective refutation information predominates, yet insufficient collective consensus triggers public resistance and distrust toward official interventions. This suggests that without the underpinning of shared public value perceptions, objective refutation efforts not only fail to alleviate public emotions but also provoke secondary public opinion risks. That is, when the power of content fails to exert positive effects and content stickiness remains weak, the positive impacts of rumor refutation diminish, thereby inducing the contagion of negative digital emotions.

### Robustness checks

To ensure the robustness of the study, the analysis was re-conducted by adjusting the threshold parameters. The consistency threshold was increased from 0.8 to 0.85, Such adjustment reveals that the mean consistency and coverage of the four configurations vary by less than 10%. In line with common criteria [85], all indicators meet robustness requirements, confirming that the research results are robust.

## Discussion

Based on the tipping point theory, this study integrates SA, SNA and fsQCA methods. From the perspectives of micro emotional attributes, meso network structure, and macro evolutionary pathways, this study analyzes 160,000 comments collected from Weibo regarding 12 typical health events. This study realizes theoretical framework integration and innovation, expands existing research perspectives, and further promotes practical application and theoretical extension of relevant theories. By applying fsQCA, this study identified equivalent configurations that drive digital emotion contagion pre- and post-refutation in online discourse. The analysis of the digital emotion contagion process is primarily grounded in the analytical framework of tipping point theory. Correspondingly, the computational results reveal how interactions among communication content, the communication environment, and communicators contribute to the emergence of positive or negative digital emotions among netizens. Furthermore, these findings highlight the differences in digital emotion contagion mechanisms pre- and post-refutation.

The primary goal of refutation rumors is to reduce their negative impact and guide netizens to view public health events rationally and comprehensively. By analyzing the conditional variables in the configuration calculation results, three models that are likely to trigger positive digital emotion among netizens can be identified.

**Fig 5:**
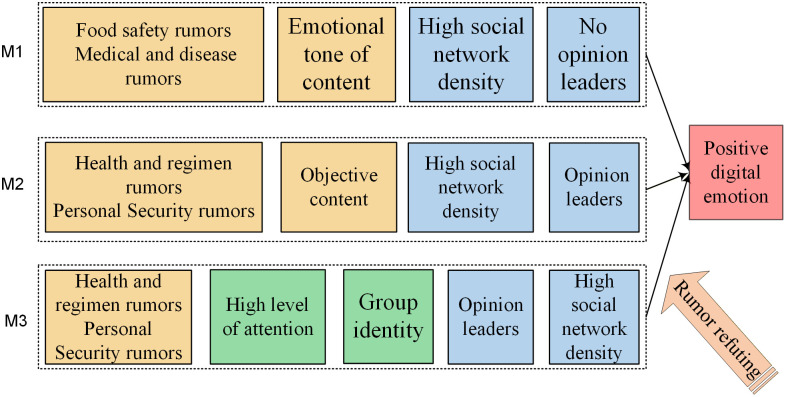
A schematic diagram of the three models of positive digital emotion contagion.

Model 1: An “information equality-type” communication model for food safety or medical and disease rumors prior to refutation. As shown in Fig 5(M1), under this model, public health topics are initially communicated on the internet without interference. Topics that involve a wide range of people are likely to resonate emotionally with netizens, who may communicate using radical language or symbols. In a scenario where discourse power is decentralized, after high density online communication, the probability of positive digital emotions emerging is relatively high.

Model 2: A “information centralization-type” communication model for topics related to health and regimen or personal security prior to rumor refutation. As shown in Fig 5(M2), when health and regimen and personal security topics are presented in objective language and disseminated by opinion leaders through high density networks, positive digital emotion contagion occurs more rapidly.

Model 3: A “information centralization-type” communication model for health and regimen or personal security topics following the rumor refutation. As shown in Fig 5(M3), this model demonstrates the positive regulatory effect of refutation content on digital emotions. After the refutation content is released, netizens participating in the discussion return to rationality. Opinion leaders retweet refutation content and exploit dense networks to spread positive digital emotions. As discussing netizens share consistent values and pay increasing attention to such content, digital emotion contagion is accelerated.

From the mechanistic perspective, such inappropriate rumor refutation can be explained by the backfire effect and motivated reasoning. After the release of rumor refutation messages, the core of user discussion shifts from factual authenticity to consistency between viewpoints and their inherent stances. Driven by motivated reasoning, some netizens tend to selectively accept information consistent with their preexisting stances, lifestyles, and group identities, thereby recoding rumor refutation content as materials for positional confrontation. This process can be further elucidated from the three dimensions of the tipping point theory. First, in line with the law of the few, the attitudes of opinion leaders toward rumor refutation content may serve as emotional signals for their followers. In particular, their confrontational expressions tend to amplify negative emotions. Second, with regard to the stickiness factor, the emotional tone of content and repetition of original rumor claims in rumor refutation texts shape the contagion intensity of such information among users. Third, from the perspective of the power of context, high network density and frequent online interactions tend to trigger the echo chamber effect and group polarization, laying the groundwork for the diffusion of negative digital emotions. The above interpretations corroborate the configuration findings that negative emotion contagion is more likely to occur following rumor refutation.

Institutional trust and information fatigue may exert significant moderating effects on the aforementioned mechanisms. Although this study does not directly measure these two variables, their values can be inferred from the configuration characteristics of emotional reversal post-refutation. In the Shuanghuanglian incident, when institutional trust is low and rumor refutation content is presented via objective argumentation, such refutation is more likely to be perceived as a tool for speech control, thus providing mobilizable rationale for the confrontational framing of opinion leaders. As public attention to this topic grows and netizens have formed prior negative perceptions toward the rumor, negative emotion contagion ultimately ensues. As information fatigue builds up, coupled with information overload and repeated corrective information exposure, users lack the motivation for in-depth analysis and tend to voice opinions driven solely by emotions. According to configuration AN3, following official rumor refutation regarding the claim that health products can cure COVID-19, frequent interactions among users with similar values tend to trigger information overload. Consequently, netizens are disinclined to engage in in-depth reasoning and careful judgment, and are more prone to voicing opinions purely on an emotional basis.

### Theoretical implications

Research findings suggest that the greater the universality of a topic, the more pronounced the effectiveness of official refutation efforts. An analysis of the structural composition of digital emotions triggered pre- and post- refutation reveals that netizens are more likely to display positive digital emotions when engaging with topics related to food safety, as well as medical and disease rumors. Topics related to health and regimen and personal security, due to their narrower audience, tend to resonate quickly and discreetly within specific groups. The digital emotions associated with such topics solidify rapidly, making it challenging to conduct timely and accurate refutation efforts.

In the communication environment, the influence of opinion leaders and network density on negative digital emotions is more pronounced. If discussions on public health topics exhibit high network density and are deeply engaged by influential opinion leaders who openly promote negative digital emotions, authoritative institutions may need to promptly and accurately refute rumor to prevent the emergence of group polarization. Configuration results indicate that the power of context within the tipping point theory better explains the emergence and diffusion of negative digital emotions in public health discussions. The configuration calculation results also highlight a special case of situational intervention: If health and regimen and personal security topics involve netizens with high group identity, and most posts are rational, then authoritative institutions may not need to intervene, as netizens may spontaneously generate and spread positive digital emotions among themselves.

The relationship between the individual characteristics of communicators and the effectiveness of rumor refutation efforts is relatively clear. For rumor refutation to exert positive effects on digital emotions, two conditions must be concurrently satisfied: First, refutation content must attract widespread public attention; second, engaged users must hold similar values. The absence of either condition undermines authoritative institutions’ rumor refutation efficacy. Configuration findings indicate that without the coexistence of public attention and group identity, positive digital emotions may spread spontaneously among internet users under favorable information communication contexts, while forced rumor refutation tends to yield adverse outcomes.

### Application implications

Food safety, medical and disease rumors are topics that attract a wide audience. If negative digital emotions arise during the initial dissemination of public opinion information without interference, then refutation information should use rational language as its medium and adopt a multi-platform release strategy to quickly convey the true situation to various online communities. Since digital emotion contagion is controlled by social media operators, operators may increase the frequency and intensity of netizens’ exposure to emotions to boost user engagement. Therefore, public management departments should actively establish social media accounts and promote rumor information. This enables netizens to maintain a rational perspective on rumors amidst a sea of emotionally charged comments. For health and regimen and personal safety topics, variations in the structural conditions of negative digital emotions pre- and post-refutation suggest that legal constraints on opinion leaders’ public opinion influence, together with public warnings against severe penalties for reckless rumor sharing, can impede rumor diffusion and mitigate negative emotion contagion in both scope and velocity. From the perspective of information recipients, public management departments aiming to use rumor refutation to encourage netizens to approach health topics with positive digital emotions should prioritize highly active users in topic interactions who demonstrate a strong sense of group identity, and promptly convey accurate information and remedial measures to these individuals.

Public health topics encompass issues that impact the basic well-being of the general public. When health related incidents spark public concern and rumors begin to circulate, relying solely on official channels to refute rumors is insufficient. Media operators, professional researchers, and public figures, as managers in the media environment, need to have a basic awareness of collaborative cooperation, establish a rational media environment for public health topics, and jointly promote the dissemination of rumor refutation information and the guidance of digital emotions.

### Limitations and prospects

In terms of research perspective, the design of the variable association model is primarily based on tipping point theory, which demonstrates excellent applicability and flexibility in studying the intrinsic patterns of social trends. Beyond the independent and dependent variables selected in this paper, the three golden rules and the critical threshold concept proposed by tipping point theory provide scholars with a framework for selecting variables across broader domains and analyzing the dissemination patterns of health topics.

In terms of variable assignment scheme design, the assignment scheme based on fuzzy mathematics membership data for topic types helps improve case coverage but cannot provide precise analysis of sub-topic types. In subsequent research, it is necessary to strictly refine the types of public health topics and conduct comparative studies on digital emotion contagion methods. The calculation of opinion leadership influence using network analysis technology has advantages in identifying opinion leaders and assessing their influence, but the semantic judgment of opinion leaders’ statements can only rely on secondary text analysis. This data processing method needs improvement in terms of execution efficiency. Additionally, the influence of opinion leaders on information dissemination can be measured from aspects such as intervention time, emotional orientation, and interaction frequency. This provides expanded research directions for future studies. Furthermore, variables including institutional trust and information fatigue proposed in the mechanistic discussion of this study currently serve merely as a theoretical framework for explaining configuration results. Future research may directly measure these psychological and cognitive variables via questionnaires or controlled experiments to further verify their exact moderating effects.

In terms of case selection, this study needs to balance topic influence, the existence of rumors, and the authority of refutation information. This results in limitations on case coverage. Such sampling neglects abundant low-impact health misinformation prevalent on social media. Accordingly, the identified equivalent configurations of digital emotion contagion in this study are more applicable to explaining sudden, high-profile public health crises. But this research limitation cannot be simply addressed by increasing the sample size. Future research on digital emotion contagion related to health topics can improve case coverage by appropriately relaxing the criteria for case selection. Additionally, variations exist in the emphasis placed on health topics across different countries, and conducting cross-cultural and cross-regional case comparisons based on these differences offers significant research potential.

## Conclusion

In the era of deep social media development, addressing the harm caused by rumors in the communication of health topics has become a critical challenge for both governmental social governance and corporate sustainable management. Although existing research has made valuable contributions to the field of health topic communication a significant research gap persists concerning the dynamic mechanisms underlying digital emotion contagion within rumor refutation interventions. This study contributes to the literature through three key innovations. First, based on the tipping point theory, this study establishes a theoretical framework, mines large-scale datasets, and focuses on authoritative rumor refutation content as analytical nodes. It further examines digital emotion contagion pre- and post-refutation to clarify underlying influence mechanisms and inform optimized refutation strategies. Second, existing studies on digital emotion contagion largely concentrate on individual-level factors. By innovatively integrating SA and SNA, this study explores multidimensional influencing factors. Using fsQCA, it identifies variable associations and systematically delineates multidimensional trajectories of digital emotion contagion via equivalent configuration analysis. Third, using data from the Weibo platform, we selected 12 cases covering four subcategories of public health topics and 160,000 user interaction data points. This study employed network analysis and deep learning algorithms to assign values to variables and explore the routes characteristics of digital emotion contagion. After calculation, 11 configurations that triggered positive and negative emotions pre- and post-refutation were classified and discussed. The study found that health topics with a narrower audience are more likely to trigger negative digital emotions. Rumor refutation by authoritative institutions does not necessarily reinforce positive digital emotions or transform negative digital emotions. By comparing configuration patterns pre- and post-refutation, this study finds that opinion leaders, topic types and social network density exert dual effects: They facilitate positive emotion contagion prior to refutation yet amplify negative emotion contagion afterward. The findings of this study not only provide a new analytical framework for understanding the differences in the routes of digital emotion contagion pre- and post-refutation but also offer valuable insights for reshaping trust mechanisms among netizens in digital media and enhancing crisis response capabilities for public sectors and businesses.

## Supporting information

S1 Table Membership scores of all variables pre-refutation. (DOC)

S2 Table Membership scores of all variables post-refutation. (DOC)

S3 Table Calibration results of all variables. (DOC)
