# Supplementary material for: Exploring the contagion routes of digital emotion on health topics: A multidimensional configuration analysis pre-post rumor refutation
Source: PLoS One. 2026 Aug 5;21(8):e0352675. doi: 10.1371/journal.pone.0352675 (PMC13440863; doi:10.1371/journal.pone.0352675)
Supplement: Supplementary file 1 [file pone.0352675.s001.doc]

| Variable name | 5% | 50% | 95% |
| --- | --- | --- | --- |
| Digital emotion | -1.24787 | -0.28596 | 1.413106 |
| Topic type | 0 | 0.5 | 1 |
| Emotional color of the post | -6.94389 | 2.22363 | 30.04110385 |
| Opinion leader | 0.000809 | 0.00881 | 0.11125 |
| Network density | 0.0015 | 0.0135 | 0.16135 |
| Attention of netizens | 119.36 | 991.65 | 22500 |
| Group identity | 0 | 0.155 | 0.464 |

Note: Digital emotion serves as the outcome variable, while all other variables are treated as antecedent variables. The 5%, 50% and 95% percentiles of sample data are defined as the threshold values of completely unaffiliated point, intersection point, and the full affiliated point, respectively.
