# Supplementary material for: Exploring the contagion routes of digital emotion on health topics: A multidimensional configuration analysis pre-post rumor refutation
Source: PLoS One. 2026 Aug 5;21(8):e0352675. doi: 10.1371/journal.pone.0352675 (PMC13440863; doi:10.1371/journal.pone.0352675)
Supplement: Supplementary file 2 [file pone.0352675.s002.doc]

| Variable name | 5% | 50% | 95% |
| --- | --- | --- | --- |
| Digital emotion | -1.3466 | 0.265148 | 5.2127469 |
| Topic type | 0 | 0.5 | 1 |
| Emotional color of the post | -5.291521 | 15.53144 | 114.786435 |
| Opinion leader | 0.0009686 | 0.005518 | 0.09505 |
| Network density | 0.001 | 0.011 | 0.1198 |
| Attention of netizens | 51.26 | 704.93335 | 501470.7525 |
| Group identity | 0 | 0.0085 | 0.06565 |

Note: The above contents are the membership scores post-refutation. The 5%, 50% and 95% percentiles of sample data are defined as the threshold values of completely unaffiliated point, intersection point, and the full affiliated point, respectively.
