# Supplementary material for: Exploring the contagion routes of digital emotion on health topics: A multidimensional configuration analysis pre-post rumor refutation
Source: PLoS One. 2026 Aug 5;21(8):e0352675. doi: 10.1371/journal.pone.0352675 (PMC13440863; doi:10.1371/journal.pone.0352675)
Supplement: Supplementary file 3 [file pone.0352675.s003.doc]

| Case | Topic Type | Emotional Color of the Post | Opinion Leader | Network Density | Attention of Netizens | Group Identity | Digital Emotion |
| --- | --- | --- | --- | --- | --- | --- | --- |
| 1 | 0.95 | 0.870181683 | 0.116663056 | 0.088586744 | 0.037283441 | 0.582613777 | 0.912084807 |
| 2 | 0.731276615 | 0.818974492 | 0.046519437 | 0.044483915 | 0.066099898 | 0.339645739 | 0.120862846 |
| 3 | 0.95 | 0.698732716 | 0.053019763 | 0.056159888 | 0.514187326 | 0.605587511 | 0.910350612 |
| 4 | 0.268723385 | 0.120629136 | 0.965764472 | 0.974281638 | 0.911201986 | 0.05 | 0.031463753 |
| 5 | 0.05 | 0.150839607 | 0.50302517 | 0.512444331 | 0.165748018 | 0.828107435 | 0.662705673 |
| 6 | 0.05 | 0.470156791 | 0.115567742 | 0.088586744 | 0.855796902 | 0.628103899 | 0.072449501 |
| 7 | 0.731276615 | 0.05142065 | 0.515731631 | 0.561915096 | 0.975811099 | 0.071457627 | 0.869809032 |
| 8 | 0.268723385 | 0.679888049 | 0.932205965 | 0.915277612 | 0.1452035 | 0.05 | 0.094955668 |
| 9 | 0.268723385 | 0.312180909 | 0.461252908 | 0.351278354 | 0.063361279 | 0.260224714 | 0.232732447 |
| 10 | 0.95 | 0.509845552 | 0.427105365 | 0.248960384 | 0.602969183 | 0.339645739 | 0.950135155 |
| 11 | 0.731276615 | 0.985483396 | 0.859522552 | 0.870104894 | 0.197818728 | 0.671432161 | 0.176516592 |
| 12 | 0.05 | 0.048314133 | 0.547455239 | 0.561915096 | 0.62614486 | 0.99025811 | 0.949889086 |

Note: The above results are the calibration of digital emotions generated before rumor refutation.

| Case | Topic Type | Emotional Color of the Post | Opinion Leader | Network Density | Attention of Netizens | Group Identity | Digital Emotion |
| --- | --- | --- | --- | --- | --- | --- | --- |
| 1 | 0.95 | 0.637901649527239 | 0.0543305658943362 | 0.0500000000000001 | 0.500052379885119 | 0.981887888077881 | 0.912084807 |
| 2 | 0.731276615084765 | 0.370529088681405 | 0.0451476820101043 | 0.0500000000000001 | 0.997510857216199 | 0.372944163194269 | 0.0455326371219933 |
| 3 | 0.95 | 0.825430861224896 | 0.0655701052800616 | 0.0659893126563793 | 0.51808750826194 | 0.619977281652058 | 0.832085784037383 |
| 4 | 0.268723384915235 | 0.229575581022651 | 0.50819635072028 | 0.527036461474952 | 0.0560176616587026 | 0.88959494302428 | 0.0539601520025572 |
| 5 | 0.0500000000000001 | 0.99047601285727 | 0.943280553421066 | 0.917480324979588 | 0.16521170038111 | 0.0500000000000001 | 0.205937468341748 |
| 6 | 0.0500000000000001 | 0.253854905295021 | 0.0986620263549152 | 0.0866255450880921 | 0.610614545081635 | 0.29608491048594 | 0.187446024416814 |
| 7 | 0.731276615084765 | 0.00628401718652645 | 0.587259397731289 | 0.567247214575294 | 0.503063179031257 | 0.51931085554738 | 0.706197889886293 |
| 8 | 0.268723384915235 | 0.517655434831437 | 0.957189977288022 | 0.964486012887446 | 0.256440565100979 | 0.0500000000000001 | 0.123863161616302 |
| 9 | 0.268723384915235 | 0.293645270941815 | 0.20092723992878 | 0.112934854032767 | 0.0434757065837717 | 0.51931085554738 | 0.969693359976427 |
| 10 | 0.95 | 0.588971060771041 | 0.427105365 | 0.248960384 | 0.602969183 | 0.0500000000000001 | 0.760411370085414 |
| 11 | 0.731276615084765 | 0.732932887069015 | 0.898506304576764 | 0.934144002964517 | 0.212404065633192 | 0.678537316364819 | 0.0673402403964418 |
| 12 | 0.0500000000000001 | 0.416595144004011 | 0.344055511631575 | 0.2354523864623 | 0.502275592772327 | 0.29608491048594 | 0.608174839993897 |

Note: The above results are the calibration of digital emotions generated after rumor refutation.
